# Adding spontaneity to organizations – what hospice volunteers contribute to everyday life in German inpatient hospice and palliative care units: a qualitative study

**DOI:** 10.1186/s12904-024-01409-3

**Published:** 2024-03-28

**Authors:** Armin Nassehi, Irmhild Saake, Christof Breitsameter, Anna Bauer, Niklas Barth, Isabell Reis

**Affiliations:** 1 Department of Sociology, Ludwig-Maximilians-Universität München, Konradstraße 6, 80801 Munich, Germany; 2 Department of Catholic Theology, Ludwig-Maximilians-Universität München, Munich, Germany

**Keywords:** Death, Dying, Inpatient hospice care, Palliative care, Volunteers, Role definition, Organizations, Health services research

## Abstract

**Background:**

Volunteers have always been integral to hospice and palliative care. However, their roles have been left relatively undefined and broad.

**Aim:**

This study aims to examine the role of hospice volunteers in German inpatient hospice and palliative care. The question we seek to answer is: What do hospice volunteers contribute to everyday life in inpatient hospice and palliative care units?

**Methods:**

We undertook a multicenter, on-site qualitative interview study, utilizing problem-centered interviews with 16 volunteers from five inpatient hospice units and one hospital palliative care unit. Interviews were analyzed using grounded theory.

**Results:**

Analysis of the interviews revealed three typical characteristics of how hospice volunteers’ describe their own role: (1) performing small acts of kindness, (2) creating a family-like atmosphere, (3) expecting emotional experiences. A common theme across all categories is the emphasis on spontaneous actions and personal experiences. The process of dying becomes an experience interpreted by volunteers as enriching, as a gift, as a “teacher”.

**Conclusion:**

Granting hospice volunteers freedom to act spontaneously and intuitively benefits hospice and palliative care delivery. Organizations should leave sufficient room for spontaneity in order to involve volunteers effectively. Open and unstandardized roles facilitate dynamic work practices.

**Supplementary Information:**

The online version contains supplementary material available at 10.1186/s12904-024-01409-3.

## Introduction

Hospice volunteers played a pivotal role in the emergence of the palliative care and hospice movement. Particularly in the early days, when specialized support and care for the dying were not yet commonly available within the established structures of health care systems, volunteers spearheaded the development of alternative care practices, procedures, and philosophies [1]. In Germany, palliative care has evolved into a professional and integral component of the health care system in recent years [2]. Despite this professionalization, hospice volunteers continue to be a part of German hospice and palliative care. As such, based on an extensive body of international research literature on hospice volunteers, the following questions may be posed: Who are hospice volunteers? How do they perceive their own role? What contributions do they make to hospice and palliative care?

International studies indicate that volunteers are commonly older, female and middle-class [3, 4]. Recent analyses in Germany corroborate these findings [5]. Numerous studies have explored the motivations behind voluntary commitments to hospice work, often finding that the death of a close relative can act as a catalyst for such volunteer work [6, 7]. The motives of volunteers oscillate between altruism and personal interests [8]; they aim to assist others, while concurrently seeking to learn and grow personally [6, 9, 10]. It’s been observed that volunteers experience ‚personal growth‘ [11] from their involvement. A recent study furnishes evidence suggesting that volunteers may enhance their psychological well-being through their activities [12].

Several international studies suggest that volunteers undertake an array of tasks within the hospice context. They assist with care, they go shopping, help with household chores, perform driving duties [11], support grieving families [13], prepare meals and beverages, read aloud, engage in board games [14], keep vigil at the bedside during the night, go for walks, conduct fundraising [15], manage the reception, run the snack bar [3], engage in casual conversations, remain simply present [16, 17], and perform miscellaneous ‘odd jobs’ [3]. From this broad range of tasks, it seems impossible to derive a generalizable role description. The enormous variety of tasks has led to debates in the literature concerning the extent to which specialization, professionalization, and standardization of volunteer roles in palliative and hospice care could be necessary and effective [15, 17–20].

This study aims to address the research gap regarding a clear role description for hospice volunteers. In doing so, it also hopes to address the question of whether standardization could potentially be beneficial.

## Background

In 2023, there were 1,500 hospice services in Germany with approximately 120,000 people engaged in voluntary hospice work [21]. Despite 95% of hospice work being funded by health insurance providers, the remaining 5% underscore the connection between hospice and civil society and are said to resist the commodification of palliative care [22]. Although German hospice care could probably do without volunteers, they explicitly stick to including them. This way, the feature of voluntary work comes to the fore and can be researched in an exemplary manner.

According to the classification scheme provided by the European Association of Palliative Care, hospice volunteers in Germany typically align with the category of ‘C volunteers’ [18]. These volunteers are characterized as local community members who donate their time to help in palliative care either by being directly involved in patient care and family support or by providing organizational assistance to the professional palliative care team. They diverge from B-volunteers since they do not have professional palliative care training themselves and are not involved in the palliative care institution beyond their volunteer work. Volunteers in Germany usually undergo basic training sessions before they participate in volunteering. The aim of this preliminary training is to promote reflection on death and dying, rather than defining and instructing a specific role of hospice volunteers [23, 24].

## Methods

### Study design

The research employs a multicentric qualitative on-site interview study design (see supplement 1). Our epistemological position is guided by the sociological assumption of differentiation of roles and contexts [25, 26]. Such a position is rooted in a constructivist conceptualization of modern society as being characterized by the emergence of different functional ‘logics’ typical of modern multidisciplinary settings [27]. This leads to the assumption that in a multi-professional organization such as a hospice unit, different roles emerge for the different professional groups, which can conceivably be understood as functional solutions to correlated problems. From this theoretical perspective, our study investigates the role assumed by volunteers and the issue that it seeks to address. Hence, this study is interested in the emergent patterns within the narratives of hospice workers’ individual perspectives. The viability of this design has already been demonstrated in a similar study focusing on pastoral care workers in hospice and palliative care [28].

### Setting

Interviews were carried out in five hospice units and two palliative care units. In Germany, volunteers are more prevalent in hospice units, which typically operate their own hospice services, organizing both inpatient and outpatient palliative care as well as coordinating volunteer activities. For this study, our focus was on volunteers working within inpatient hospice services. When selecting the participating institutions, we attempted to cover different regions to account for regional variations (North, South, East, West). The inclusion criterion for participants in this study was serving as a hospice volunteer with frequent patient contact. Excluded were hospice volunteers with no contact to patients, who only handled administrative tasks. As described above, hospice volunteers with no contact to patients are relatively uncommon in Germany.

To identify characteristics typical for the role, the sample needed to maintain homogeneity, hence our focus on inpatient hospice services. For the recruitment of interviewees, the project was presented during the multi-professional team meetings, in the course of which relevant participants were invited to take part. There were no interactions with the interview partners prior to the study. Opt-outs were infrequent, and primarily due to scheduling conflicts.

### Data collection

Data were collected via problem-centered interviews (see Table 1) [29]. Most of the interviews were conducted in-person at the hospice units between 2018 and 2019 by NB, KM and AW. Interviewees did not know the interviewers beforehand. The interviews were digitally recorded and ranged from 40 to 80 min. No one else was present during the interviews. There were no follow-up interviews and the participants were not provided transcripts of the interviews. In addition to the interviews, the project members attended multi-professional team and handover meetings, during which they compiled observation records. Recruitment was ended according to our pre-established project phases.

**Table 1:** Problem centered interview protocol

**Introduction**	• Would you please start by briefly introducing yourself?
	• How long have you been working in a hospice/palliative care unit? Why did you want to work here? Have your expectations been fulfilled?
**Activity**	• What is your daily routine like? What is particularly important to you in your work?
	• Are there any situations that move you or are of particular concern to you?
	• Are there any problematic or unusual situations?
	• Did you do anything differently in the past than you do today?
	• Are there differences between what you do and what your colleagues do?
	• Do you encounter communication or moral boundaries in your work?
**Relationship with guests/patients and relatives**	• When you talk to guests/patients, what are the main issues?
	• Do the guests/patients have special needs? If so, how can you respond to the guests’/patients’ wishes?
	• How do you relate to the relatives in your daily work? Do you have the opportunity to communicate with them? How does this influence your work?
**‘Good Dying’**	• What would be successful end-of-life care for you? Can you describe a case?
	• Do you have any special wishes for the organization of the hospice/palliative care unit? What would you change?

### Analysis

**Fig. 1:**
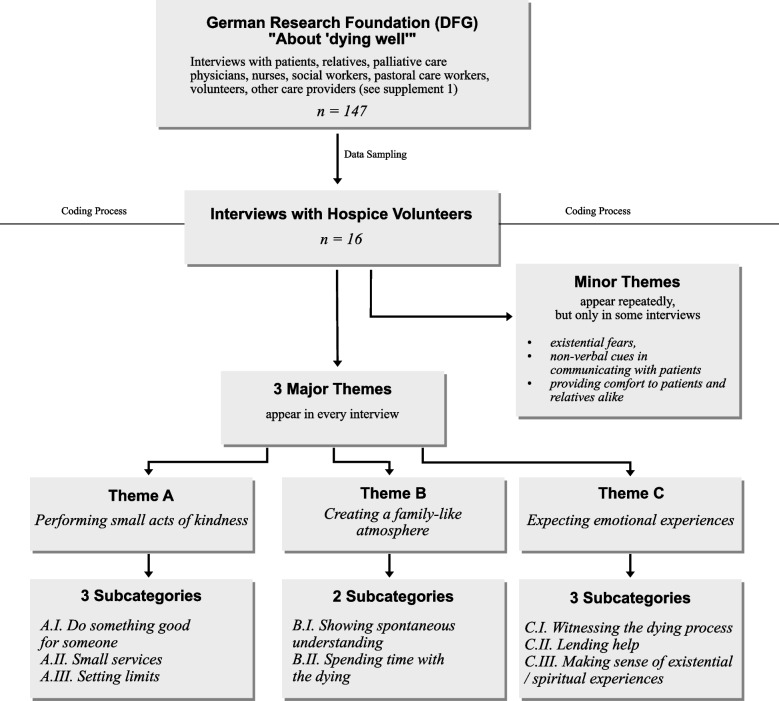
Flowchart of the research process

Interview transcripts were analyzed utilizing MAXQDA 2022. The coding process was based on the grounded theory approach, outlined by Przyborski & Wohlraab-Sahr [30]. The coding process consisted of two stages: The initial stage was an exploratory reading of the interviews, freely coding the material (open coding). Subsequently, the different codes were condensed into three to five major themes and corresponding subcategories (axial coding). A major theme was defined as a topic mentioned in every interview. For instance, witnessing the dying process was a subject discussed by all hospice volunteers, thereby solidifying it as a major theme. Minor themes also were coded. Although these themes were mentioned repeatedly, they did not appear in all interviews. The coding process saw continuous refinement and adjustment of the themes and subcategories. Table 4 presents the final coding tree. Figure 1 depicts the research process. The analysis was supplemented by recurring data sessions within the research team (see supplement 2 for more information about the team members). There was no feedback on the results from the participants in the study.

### Ethical issues

The study was approved by the ethics commission of the Medical Faculty of LMU Munich (Az-558-15, 30.11.2017). All participants were provided with an information sheet both on the project and on data protection. All participants confirmed in writing that they were willing to participate in the study.

## Results

A total of 16 hospice volunteers participated in the study (see Table 2). Interviews were conducted at six different institutions (see Table 3). At one palliative care unit we were unable to find any volunteers to interview due to two main reasons: (1) Hospice volunteers are relatively uncommon at hospital palliative care units. (2) The specific palliative care unit was located in East Germany (Saxony-Anhalt, former GDR). For historical reasons related to work organization during state socialism, hospice volunteers in this area are still less prevalent than in West Germany. Since our data is referring to the main study (see supplement 1) and we initially expected to be able to interview hospice volunteers, we deem it essential to mention the second palliative care unit.

**Table 2:** Demographic details and employment status of participants

Identifier	Age (years)	Gender	Experience (years)	Employment status
*P1*	76	F	10	Retired
*P2*	75	F	14	Retired
*P3*	19	M	0.75	FSJ (Voluntary Social Year)
*P4*	70	M	5.5	Retired
*P5*	52	F	2	No response
*P6*	73	F	10	Retired
*P7*	37	M	8	Employed (fulltime)
*P8*	59	F	9	Employed (fulltime)
*P9*	75	F	10	Retired
*P10*	45	F	3	Employed (parttime)
*P11*	62	F	5	No response
*P12*	65	F	8	Retired
*P13*	77	M	5.5	Retired
*P14*	70	M	5	Retired
*P15*	66	F	11	No response
*P16*	49	F	5	Employed (fulltime)
16 Participants	Median: 65.5	68% Female	Mean: 6.28

**Table 3:** Details of participating institutions

Identifier	# of Participants	Identitifiers Participants	Federal State (Region)	Religious affiliation
Hospice Unit 1	3	*P1, P2, P3*	Bavaria	Catholic
Hospice Unit 2	2	*P4, P5*	Lower Saxony	None
Hospice Unit 3	3	*P13, P14, P15*	Lower Saxony	None
Hospice Unit 4	4	*P9, P10, P11, P12*	Baden-Württemberg	Catholic
Hospice Unit 5	3	*P6, P7, P8*	Bavaria	Catholic
Palliative Care Unit 1	1	*P16*	Bavaria	Catholic
Palliative Care Unit 2	0		Saxony-Anhalt	Catholic

In order to analyze the role of hospice volunteers, several major themes and subcategories were derived from the data (see Fig. 1). To better illustrate our findings, we selected especially striking passages for presentation (see Table 4).

### Themes

Three major themes (categories) emerged from the data analysis, each with several corresponding subcategories (see Table 4). Minor themes, which came up frequently, but not in all interviews, revolved around talking about existential fears, the role of non-verbal cues in the communication with patients as well as providing comfort to patients and relatives alike. As this study is primarily interested in patterns that are typical of a certain perspective, these minor themes will not be discussed further.

**Table 4:** Themes (Bold) and their subcategories (Italics) reflecting the perspectives of hospice volunteers on their own role and duties in palliative care work, with illustrative quotes

Themes and Subcategories	Illustrative Quotes
**Theme A. Performing small acts of kindness**	
* A.I. Do something good for someone*	“So our task, I’ll put it very casually now, is to entertain people, to be there. To have time to do what is good for them at the moment. If you have a special request, I don’t know, would like an iced coffee, to organize that or something like that.” (P2, 29, female)
	„And if people want to do anything, then we offer that as hospice companions. Or fetching a newspaper or going shopping once, the opportunity to go together to Stefansplatz and there see what shopping with accompaniment at Tengelmann or Rossmann, for example, does. Or I was also with a lady who lives nearby, I went to her house, she wanted to see her apartment once more, so we drove home. We just provide these services, and we do that, namely when we are present in the morning.“ (P16, 15, female)
* A.II. Small services*	“There’s no [structured] process, what’s part of it here is loading the dishwasher, unloading it, sometimes it’s switching on the washing machine, putting things into the dryer, but all this happens rather casually, or it’s making a cup of coffee for a visitor.” (P10, 14, female)
	„Well, you know, after all I fulfill tasks which don’t require any training. That’s not meant to say that all I do is making coffee but that I do things like preparing breakfast for the people.“ (P3, 7, male)
* A.III. Setting limits*	„That’s an extremely demanding behavior. It’s my turn now. Of all, the man I used to go to the Lotto all the time, for example, it was him who felt offended if I didn’t devote my full attention to him. […] And then, he started driving around in his electric wheelchair, and he drove against this, and he drove against that, he was ringing the bell ten times and so. Like a spoiled little child. And then you may, and I checked back on this: then I may say: Now it’s enough. You’ve had your meal, you’ve been to the loo, you’ve had your drink. And now it’s enough.“ (P11, 56, female)
	„They just can‘t really cope with it. And there I do sometimes get, well, I‘m not very easily upset, but that‘s when I really reach my limits. When someone is so ungrateful and then still makes such demands and everything is bad. And then you really reach your limits.“ (P3, 37, male)
**Theme B. Creating a family-like atmosphere**	
* B.I. Showing spontaneous under-standing*	“For me, this is the gravest of all situations. Just being there then, watching, not doing anything. You’ve got to learn this. Many of our colleagues believe that now they must do something, but often this is impossible. And even for me this is a very grave situation. […] Or also if, and this is often the case with brain tumors, if this person isn’t all there any longer when talking. You know, if he is just talking nonsense or, however, still wants a conversation. How are you supposed to handle this?“ (P1, 19, female)
	„And then we go into the rooms and look… And there I really sensed that there is such a thing as communication without words. And you immediately notice whether someone likes being touched or not. So even if you can no longer really communicate, there are clear signals about what is good for the person and what is not. And well: Of course, I spend a bit longer in rooms with people that I haven‘t seen the week before, and try to establish contact there. Also to explain a bit about our role, because I always say what we can do and that they have to help us with it because we can‘t see inside, but especially that they are the main characters and they decide what / […] So, yes: what do I do there? I am there.“ (P2, 11, female)
* B.II. Spending time with the dying*	„Well, and I spent very much time with Mr. T. […]. Mostly I brought him supper and spent time with him. And he hardly ate, because he was talking, which I liked very much, however […] he impressed me very much. And also, the story all around, you know, which I’m not the person to judge on, fortunately, but this amiable old-aged person impressed me very much.“ (P7, 16, male)
	“Yes, it is like that, when I go into the room, I first look at the family pictures and take a good look at the room, how it is furnished, and so on. And also partly, are there lots of flowers? Then, then you can also ask if they perhaps once had a garden, right? And there are various clues…even in the room furnishings, I find things that one can address, right? Or there are also some here, like we had a post office worker, who had done many trips and then of course he talks a lot… you can get him to talk about his travels and even brought a laptop. And then about the travels…he reported wonderfully.” (P4, 18, male)
**Theme C. Expecting emotional experiences**	
* C.I. Witnessing the dying process*	“It’s very rare that we’re around right at the moment of death. Often dying takes a very long time, hours. […] But being around right at the moment of death, that’s also a piece of good luck, and I was lucky one time. Together with a very experienced nurse I helped with nursing, an old, aged lady. […] She died in my arms. Of course, that was a really, really great experience […] I never had it again. A very emotional experience. Experiencing death this way, so close …” (P1, 15, female)
	“You can simply see it in people when you see them after they have passed and you look at a person. Then you can quite well discern whether / There are some who seem as if they had seen something very beautiful. Or as if they would see something very beautiful or something / utterly relieved. And there are those who still look quite grim and stressed. And that’s why I often ask myself: What could death be like? What is it, what do they see?” (P11, 20, female)
* C.II. Lending help*	“I still remember: It was when I was with Veronika, a man passed away here, it was a summer, a few years ago, in the old hospice, he also had breast cancer. Men can also get that. And it was quite widescale. The chest area and the arm, all the way down. And he passed away in the middle of the / I had done night shifts with Veronika two or three times. And he died in the night and Veronika said: You must help me now. And then I helped her. And it was / And she always said: Are you okay? Are you okay? I said: Yes, it’s okay. And then he was dressed, everything was bandaged again and then something long-sleeved was put over it. And then she said: You have helped me a lot now.” (P11, 48, female)
	„But, well, if it happens, if it’s so terminal, being allowed to sit there then, holding the hand, perhaps lending help in some way. Once a patient even died in my arms, that’s, that’s successful palliative care. But it’s also a gift. That’s, there’s no formula.“ (P7, 75, male)
* C.III. Making sense of existential / spiritual experiences*	„Well, this just by the way, you know what I see is, and I’ve already said so, that sometimes the dying, and particularly this one I’m talking about, are really teachers for all of us. And that’s very special. And they are aware of it. Then I really address this, and I say: Have you understood what’s going on, after all? He’s our teacher. Although we’re the nursing staff or we serve them, but we’ve been made a great gift. That was a very particular soul.“ (P9, 34, female)
	“I asked her, what is it like when you think about dying? I could ask it casually. She looked at me and said, ‘I look forward to it.’ I was a bit taken aback. ‘Yes,’ she said, ‘I look forward to it. Maybe I will see my…’ she hadn’t any grandchildren, ‘my cousin or relatives again.’ And that was something that fascinated me. I always pass on this sentence, whenever the conversation comes up again, also with those who are dying. What is it like that? A resident once told me she was looking forward to it and she really died that way. I saw her when she was dying. And then she told me and I found it very great. ‘My doctor was here,’ and he probably knew that he had messed up, as you would say. ‘I forgave him.’ I found that to be a great sentence as well. So these two sentences, they will stick with me and I will always remember them from this woman.” (P1, 11, female)

### Performing small acts of kindness (Theme A)

Unlike other professional groups in palliative care, the daily work of volunteers does not adhere to a strict structure. Processes and tasks are not predefined but arise spontaneously, depending on what “is on” (P9, 9, female). However, the tasks of hospice volunteers’ are not completely arbitrary; certain patterns emerge. Some tasks, like preparing lunch or breakfast, are loosely associated with a certain time of the day. Typically, volunteers concentrate on small services which do not require specific qualifications (Table 4, A.II.). They do not care much about what they do but rather just about being able to contribute something in some way.

"It’s my task, or that’s,- what’s it like here, what’s common here, or, I’d like to say/ how do I say this? I experience myself the way I am. As a maid-of-all-work. And so, I absolutely don’t care about what I’m doing…"(P9, 8, female).

"Well, you know, after all I fulfill tasks which don’t require any training. That’s not meant to say that all I do is making coffee but that I do things like preparing breakfast for the people." (P3, 7, male).

Volunteers might also support the nursing staff with their daily tasks. One main objective repeatedly mentioned was just doing something good for someone (Table 4, A.I.). Typically they focus on patients’ wishes and desires that can’t be readily catered to within organizational routines or on tasks that may have possibly been overlooked by other staff members. An example might be the special preparation of “iced coffee” for a patient. Yet, volunteers acknowledge that there are limitations to their activities (Table 4, A.III.). Given their completely volunteer nature, they hold the power of choosing which hospice resident to assist, and which they opt not to. They can set boundaries according to their own criteria.

"And [he] was really very, well, nasty to everybody. Or to many, you know? And then, at some time I started setting limits to him and said: You know, what I’m doing here is volunteer work. And I’m not going to let you treat me like this. Well, that was a situation taking me to my limits for once, you know?" (P2, 31, female).

The fact that volunteers may decide on their own which patients they work with emphasizes the voluntary nature of their activity and thereby their differentiation from regular care practice. Hospice residents deemed overly demanding or poorly behaved (“like a spoiled little child”, Table 4, A.III.) can be sidestepped. However, this freedom to choose as well as establish one’s own limits, inherent to voluntary hospice work, is owed to a division of labor with the non-voluntary professional staff. The latter group, who are not completely free to choose their tasks as well as their preferred hospice residents, consistently undertakes all other essential services, particularly nursing and symptom control.

### Creating a family-like atmosphere (Theme B)

The second category further illustrates the intrinsically unpredictable nature of volunteer work in comparison to other roles within organizations. The hospice volunteers in our study show something we call ‘spontaneous understanding’. They may react “really intuitively” (P2, 23, female).

"And then we phoned her, and she came and was extremely upset, and she felt somehow, well, so overwhelmed […] So I just, and it simply happened, you know, then I react really intuitively, and I just hugged her and told her that she had done everything right. After all, she set her mother free. […] Well, such situations are frequent, yes. But it’s not like me mentioning things myself. It’s rather, well, it’s very much simply listening and being there. […] And being there really helps them. Somehow, it’s a family-like, emotional environment." (P2, 23, female).

This fosters a communication approach which allows volunteers to be personal and intimate in their interactions with patients while maintaining a casual tone (Table 4, B.II.). The way volunteers manage interactions with relatives underscores their ability to react situationally, intuitively, and spontaneously – in their respect – in emotionally appropriate ways. In doing so, they cultivate a somewhat familial atmosphere within the hospice unit.

Another common theme drawn from the interviews revolves around the act of simply being present and devoting time to a resident during the dying process, even if communication is no longer possible (Table 4, B.I.).

"For me, this is the hardest of all situations. Just being there then, watching, not doing anything. You’ve got to learn this. Many of our colleagues believe that now they have to do something, but often it’s just impossible. And even for me this is a very grave situation. […] Or if, and this is often the case with brain tumors, if this person isn’t at all present any longer when talking. You know, if he is just talking nonsense or, however, still wants a conversation. How are you supposed to handle this?" (P1, 19, female).

Maintaining a personal relationship, even when the dying individual ceases to respond, is often viewed as a significant achievement. Even when there is nothing left to do, hospice volunteers can continue their bedside presence. In organizations, it can be quite challenging to just sit there without doing anything, since doing nothing could be misconstrued as laziness. It is more likely for family members than for nursing staff, to be perceived as justifiably sitting by the bedside without further action.

### Expecting emotional experiences (Theme C)

Hospice volunteers follow the dying process very closely. They often attribute great significance to the direct experience of death and the dying process (Table 4, C.I.). Actually witnessing the moment of death happens mostly by chance. When volunteers are present during a person’s final moments, they typically regard it as a ‘gift’ or as ‘good luck’.

“It’s very rare that we’re around right at the moment of death. Often dying takes a very long time, hours. […] But being around right at the moment of death, that’s also a piece of good luck, and I was lucky one time. Together with a very experienced nurse I helped with nursing an old, aged lady. […] She died in my arms. Of course, that was a really, really great experience […] I never had it again. A very moving experience. Experiencing death this way, so close …” (P1, 15, female).

This profound, almost spiritual experience of being very close to death while sitting at the bedside is intensified by the volunteers’ perception of their role as supportive to the nursing staff (Table 4, C.II.). In their capacity as volunteers, they can sit at the bedside and accompany the dying and thereby alleviate the nursing staff’s workload. Witnessing the dying process – and more significantly, doing so while providing practical help to nurses – represents a critical objective for volunteers. It affords a sense of involvement in the dying process that creates proximity to those who are in the final stages of their life.

However, from the point of view of the volunteers, one can get even closer. Volunteers frequently portray death and the preceding dying process, which can easily become routine in hospice care, as exceptionally special experiences (Table 4, C.III.). The dying individual might even evolve into a kind of existential figure.

"Well, just by the way, you know what I see is, and I’ve already said this, that sometimes the dying, and particularly this one I’m talking about, are really teachers for all of us. And that’s very special. And they are aware of it. Then I really address this, and I say: Have you understood what’s going on, after all? He’s our teacher. Although we’re the nursing staff or we serve them, but we’ve been given a great gift. That was a very special soul." (P9, 34, female).

For volunteers, dying is something exceptional, an intimate and individual moment during which they may come particularly close to death. The volunteer cited here describes the dying person as a kind of teacher, educating her about mortality in general and the finitude of life. For her, dying is far from being routine. Despite how unusual it seems that the volunteers mostly focus on their own experiences, it is precisely in doing this that they perform the role of someone who is part of an organization, yet simultaneously distancing themselves from it. For them, death is viewed as a gift, a teacher imparting understanding of life and death. Their willingness to perceive interactions with the dying person as enriching personal experiences contrasts significantly with the routinized approach to death that is inevitable within professional organizations.

## Discussion

### Main findings of the study

The findings highlight that hospice volunteers often engage in small acts of kindness that do not necessitate any specific skills or expertise. They participate in casual conversations with patients and relatives, and closely observe dying individuals while sitting at the bedside. At first glance, these actions do not appear to be extraordinary as they could equally be performed by other hospice care staff. However, it is striking how these task descriptions are more commonly attributed to volunteers than other professional groups.

What aspects, then, are unique about volunteer work at inpatient hospice care? What role can be derived from this? Our analysis indicates that the traits articulated in relevant literature, such as a wide range of activities [3] as well as motivation beyond personal interests [8–11], stem primarily from a role that is distinctly characterized by being different from organizational rules and routines. While volunteers perform expected tasks, they also have the latitude – at least in the German care context – to selectively accept or reject these tasks [15].

This specific organizational ‘anomaly’ makes spontaneous reactions predictable, producing a form of communication evocative of a familial setting. Actions such as unexpectedly hugging someone, responding emotionally or just being present [18, 31] contribute elements that are difficult to organize. Simply sitting by the bedside and accompanying the dying is another genuine form of support in a daily routine where professional caregivers are often preoccupied.

All of this is justified and motivated by a very personal intention—to come as close as possible to death, thereby gaining a deeper understanding of it. This describes the fundamental nature of volunteers’ assistance in this field. There is an aspect of spontaneity once again, this time associated with death, which grants volunteers a profound experience.

The benefit for the organization is that spontaneous elements disrupt a routinized daily life. In inpatient hospice care, routines pose a particular challenge as they render death and dying predictable, subjecting them to trivialization [32]. Volunteer hospice workers succeed in viewing death as an unexpected gift, understand it as a teacher, and as such, attribute significance that transcends the immediate circumstances.

We consider these results to be transferable to other countries with much more defined job roles for volunteer hospice workers. The more specialized and formalized the unpaid activity, the more it needs to align with the personal interests of volunteers. In this context, the role, as compared to other employees, stabilizes as one that partially deviates from routines, thus allowing room for personal engagement. The desire to come as close as possible to death and dying, and to be emotionally moved by it, is a common theme in international literature, as well as in the German context [33].

### What this study contributes

This study adds insights into the role of hospice volunteers. The results of the study underpin the objective, articulated in the European Association of Palliative Care White Paper, to identify the role of volunteers as ‘being there’ in line with the Asclepian tradition [18, 31]. Our data also pinpoints ‘being there’ as a key element in how volunteers describe themselves. Simply through being present, volunteers find opportunities to make spontaneous contributions in their everyday life by offering casual assistance, thereby fostering a familial atmosphere.

In line with previous literature, this study shows that the tasks of volunteers are heterogeneous. While certain studies have proposed that a higher degree of standardization might support volunteers with more confidence in their actions due to a more clearly defined role [11, 17, 18], our findings do not support this assertion. Recent studies cautioned that an overly rigid regulation of the tasks of volunteers might support an unwanted ‘them and us’ mentality between volunteers and the other staff [15], no longer making volunteers as visible as the other, more accessible ‘face’ of nursing [19]. Another recent study revealed that standardization efforts have relatively little impact on volunteering practices [20]. Our study’s results do not reveal such a “them and us” mentality nor do they enable any conclusions regarding the effectiveness of specific standardization efforts.

However, our observations did reveal a crucial conclusion – and this is the most important conclusion to be drawn from our study – that even in the absence of notable standardization efforts, the somewhat ‘unstandardized’ and spontaneous behavior of hospice volunteers has effectively become a standard within German hospice care.

From a sociological point of view, volunteers, owing to the unspecific definition of their role, present a kind of ‘anomaly’. As members of the organization, they contradict everything that usually shapes an organization: their training is not specific, their actions are mostly spontaneous, they devote their personal time to hospice work, and they work without being paid. Yet, this status of an ‘organizational anomaly’ comes about only in the presence of an ‘organizational normality’ that fosters continuity and predictability. In this light, the apparent randomness of the volunteers’ tasks could be seen as an effect of the division of labor within multi-professional hospice care, which they are part of. This way, they can be described as an ‘expectable anomaly’.

Modern palliative care has faced criticism for being subjected to a process of “creeping medicalization” due to professionalization and specialization [34]. However, our research illuminates that hospice volunteers foster a relaxed, familial atmosphere, infusing the hospice unit with a touch of ‘normality’ [35]. Consequently, it can be deemed a ‘counter-institution’ [4], according to its historically rooted self-understanding as described at the beginning of this study. It is worth noting that this is not accomplished in opposition to the institution, but rather through institutional and organizational methods.

### Strengths and weaknesses of the study

The strength of this study is rooted in its design, based on the sociological assumption of differentiating contexts and roles [25, 26]. As a result of the number of interviews conducted, it was possible to identify a variety of different categories and subcategories. There are limitations due to the focus on Germany. Since only one volunteer working at a palliative care unit was interviewed, the study does not allow for stating in how far the activities of volunteers at hospital palliative care units are different from those at hospice units. This is an interesting topic for future research. A possible future research direction could be how policies might potentially negatively impact this spontaneity or further amplify its benefits. Moreover, we suggest the potential for future research to evaluate the impact of hospice volunteers’ spontaneous work on the emotional well-being of patients and relatives.

As an implication for practical work, we suggest that hospice managers should value the benefits of unstandardized roles and structures as they facilitate dynamic and flexible work practices.

## Conclusion

The findings imply that allowing spontaneity to volunteers can be advantageous. Organizations delivering hospice and palliative care should provide these spaces if they aim to involve volunteers effectively. It is also apparent that organizations like inpatient hospices and palliative care units can be adversely impacted by their own organizational formalities. The perspective of volunteers can add depth to the ‘holistic’ self-descriptions of a family-like context. However, such descriptions should not obscure the understanding of the collaborative organizational structures, which are characterized by a division of labor.

### Supplementary Information

**Supplementary Material 1.**

**Supplementary Material 2.**

## Data Availability

The coded interview material is available from the corresponding author on reasonable request.
