# Supplementary material for: Adding spontaneity to organizations – what hospice volunteers contribute to everyday life in German inpatient hospice and palliative care units: a qualitative study
Source: BMC Palliat Care. 2024 Mar 28;23:81. doi: 10.1186/s12904-024-01409-3 (PMC10976705; doi:10.1186/s12904-024-01409-3)
Supplement: Supplementary file 1 — Supplementary Material 1. [file 12904_2024_1409_MOESM1_ESM.pdf]

Supplement 1

Research Question and the German Research Foundation (DFG) Project: About ‘dying well’. Actor constellations, normative patterns, different perspectives

The importance of hospice and palliative care was politically affirmed in Germany on November 5, 2015, by the law for more extensive hospice and palliative care. This is an expression of a trend, over the past few decades, which focused on successful dying through hospice movement, stationary and ambulatory hospice care, and palliative nursing and medicine, asking the question of 'good dying.' A 'good death' now includes intricate management that incorporates medicine, pastoral care, nursing, family, and friends, with the most important aspect being each individual's encouragement to be the master of their own death. This implies that the quality of life in the final days, personal well-being, closeness to other people, as well as the extent of fears, sadness, and depression, increasingly come into focus during everyday practice of dying care and involve all participants, the affected, family and friends, caregivers, doctors, and volunteers. This broadened the perspective on the dying process, involving relatives, professionals, etc., requiring a shift from definitive solutions to perspective differences and actor constellations regarding the dying process. Sociologist Tony Walter called this new constellation of dying "neo-modern." However, these multiple perspectives can cause uncertainties in practice concerning the interpretation of a normative ideal of 'good dying.'

The research project aims to capture various perspectives and actor constellations using qualitative methods - interviews, non-participant observation, document analysis - representing a novelty in so far as the diversity of perspectives in multiprofessional organizational settings concerning dying care remains largely unexplored.

Our goal is, 1. to capture the different perspectives and actor constellations of patients/guests, professional groups, and relatives concerning dying care through qualitative research in stationary hospices and palliative care wards, and in this regard, also to consider what is understood in each case by 'living to the last' and a 'good dying,' 2. to explain the normative concepts associated with these perspective differences in their difference and similarity, and 3. on this basis, to make visible what variants a modern practice of dying empirically points to, detached from its idealized concepts. It's not about devaluing the ideal of a 'good dying,' but to describe the complexity of horizons of expectation of societal organization, institutionalization, and the discursivation of dying that the actors involved can't escape. A functional analysis of the perspective differences will contribute to understanding contemporary dying practices and has societal relevance, as it reveals the actors' actions and views and makes the needs of the dying visible. The goal is to look for signs of contingency of these processes in the practice of dying, from both the perspective of Catholic theology and sociology.

The expected results of the project could help to inform the involved actors about unobserved assumptions of their work. Accordingly, we will reveal our results to the participating institutions and discuss with them. We will then also make the results available to other healthcare institutions. Far beyond the specific research project, the project can also contribute to making a practical contribution by ensuring that

those who design palliative care wards and hospices take seriously the question of perspective difference and the complexity-increasing effect of various actor constellations. It's about clarifying how professional perspectives change in a society where it's no longer just possible to apply expert knowledge and clarity created by expertise to practical areas. Only then will the – also multiperspectival, but certainly diverse – needs of the dying become more visible. It's also about disclosing those mechanisms that inadvertently reestablish overly prescriptive and paternalistic standards with the remoralization of the topic.

Study Design

The work program includes qualitative research conducted in hospices and palliative care wards. Our research was designed as a multicenter, on-site qualitative interview study.

More information is available on our website: https://www.gutessterben.uni-muenchen.de/index.html
