# Supplementary material for: Adding spontaneity to organizations – what hospice volunteers contribute to everyday life in German inpatient hospice and palliative care units: a qualitative study
Source: BMC Palliat Care. 2024 Mar 28;23:81. doi: 10.1186/s12904-024-01409-3 (PMC10976705; doi:10.1186/s12904-024-01409-3)
Supplement: Supplementary file 2 — Supplementary Material 2. [file 12904_2024_1409_MOESM2_ESM.pdf]

Supplement 2

Researcher position at the time of the study

Identifier	Function	Occupation	Gender	Credentials	Abbrev.
Researcher 1	Project Manager, data analysis	Full professor sociology, PhD in Sociology	m	PhD, Prof.	AN
Researcher 2	Project Manager	Full professor moral theology, PhD in Theology	m	PhD, Prof.	CB
Researcher 3	Project Manager, data analysis	Senior Researcher, PhD in Sociology	w	PhD	IS
Researcher 4	Interviews, data analysis	Research associate, Diploma in Sociology	m	Dipl.-Soz.	NB
Researcher 5	Interviews, data analysis	Research associate, Diploma in Sociology	w	Dipl.-Soz.	KM
Researcher 6	Interviews, project management	Research associate (2014-2020), PhD in Philosophy, Master of Medicine, Ethics and Law	m	PhD, M.mel.	AW
Researcher 7	Data analysis, writing	Student Research Assistant (since 2019), Bachelor in Educational Sciences	w	BA	IR
Researcher 8	Data analysis. writing	Research associate (since 2017), Master of Sociology	w	MA	AB
